# Evaluation of biventricular function by cadmium–zinc–telluride SPECT gated tomographic radionuclide angiography: Comparison to conventional SPECT

**DOI:** 10.1097/MD.0000000000039821

**Published:** 2024-09-27

**Authors:** Yue Chen, Zekun Pang, Jiao Wang, Xuewen Yang, Jianming Li

**Affiliations:** a Department of Nuclear Medicine, TEDA International Cardiovascular Hospital, Tianjin, China.

**Keywords:** biventricular function, cadmium–zinc–telluride, CZT, equilibrium radionuclide ventriculography, ERNV, repeatability, single-photon emission tomography, SPECT

## Abstract

We compared and analyzed the consistency and repeatability of left and right ventricular ((LV/RV) functions obtained by gated-equilibrium radionuclide ventriculography (ERNV) with cadmium–zinc–telluride single-photon emission computed tomography (CZT-SPECT) and conventional SPECT (C-SPECT) with sodium iodide crystal detectors. Seventy-seven patients were included in the retrospective study. Both C-SPECT and CZT-SPECT imaging were performed on the same day. Correlations and differences in LV/RV ejection fraction (LVEF and RVEF), peak ejection rate (PER), and peak filling rate (PFR) were compared between the 2 models. Cardiac magnetic resonance (CMR) was partially used as the gold standard, and ultrasound results were included for comparative analysis. Interobserver reproducibility of each parameter obtained by the 2 cameras was compared. Between the 2 cameras, there were no significant difference in LVEF, LVPER, LVPFR, and RVPER (*P* > .05) and there were in RVEF and RVPFR (*P* < .05 or .001). The correlations (*R* value) were 0.831 (LVEF, excellent), 0.619 (RVEF, good), 0.672 (LVPER, good), 0.700 (LVPFR, good), 0.463 (RVPER, normal), and 0.253 (RVPFR, poor). There were no significant difference between CMR and CZT-SPECT in LVEF (*P* > .05) while there were between CMR and both C-SPECT and ultrasound (*P* < .05). The correlations were all good (*R* = 0.660, 0.658, and 0.695). There were no significant difference between CMR and both C-SPECT and CZT-SPET in RVEF (*P* > .05) and the correlations were good (*R* = 0.771 and 0.745). For repeatability, the intraclass correlation coefficient of RVPFR by C-SPECT was good (intraclass correlation coefficient = 0.698) and excellent for the rest of the groups (0.823–0.989). The repeatability of LVEF and RVEF was better for CZT-SPECT than for C-SPECT. The repeatability of PER was better for both cameras than PFR. CZT-SPECT tomographic ERNV correlated well with C-SPECT planar ERNV in evaluation of biventricular systolic function and LV diastolic function. Compared with the “gold standard” CMR, both models had good correlation in measuring LV/RVEF. CZT-SPECT had better inter-group reproducibility than C-SPECT. The accuracy of RV diastolic function need further study. CZT-SPECT tomographic ERNV will play an important and unique role in the clinical application of accurate evaluation of biventricular function in the future.

## 1. Introduction

Gated-equilibrium radionuclide ventriculography (ERNV) is able to noninvasively reflect functional parameters of the right and left ventricles (LV/RV), which is very useful for the evaluation of coronary artery disease, cardiac conduction, and the auxiliary diagnosis of cardiomyopathy. It is important to monitor the cardiotoxic side effects of chemotherapy and targeted therapy in non-cardiovascular diseases, especially in cancer patients.^[1,2]^ The assessment of synchronization of LV/RV mechanical contraction is also gaining clinical attention.^[3,4]^ Planar ERNV was widely used in clinic, but atrial and ventricular overlap, background outlining and count rate can affect its accuracy in the determination of ventricular function, especially the accuracy of ventricular volume measurement.^[5,6]^ Tomographic ERNV has overcome these problems and have better agreement and correlation with cardiac magnetic resonance (CMR) results.^[7,8]^ It has become the first alternative examination for accurate assessment of LV/RV conditions in patients with metal implants (contraindication to CMR).^[9]^ However, temporal, spatial, and energy resolution, as well as counting sensitivity have been challenges limiting the diagnostic efficacy of nuclear medicine imaging. Cadmium–zinc–telluride (CZT)-single-photon emission computed tomography (SPECT) has achieved a great improvement in the above-mentioned performance.^[10]^ The high frame rate, low-dose tomography has greatly improved acquisition performance of ERNV.^[11,12]^ CZT-SPECT tomographic ERNV has been less studied in recent years due to the limitations of equipment and technology availability. But the existing studies on its diagnostic efficacy and acquisition protocol optimization have shown the potential value of this technology.^[13,14]^

In this study, we analyze the consistency of LV/RV function obtained by ERNV with CZT-SPECT tomography and conventional SPECT (C-SPECT) planar. And we also compare the interobserver reproducibility of various functional parameters obtained with both cameras. The purpose is to explore value of the CZT-SPECT tomographic ERNV for the evaluation of cardiac biventricular function.

## 2. Materials and methods

### 2.1. Study population

**Figure 1.**
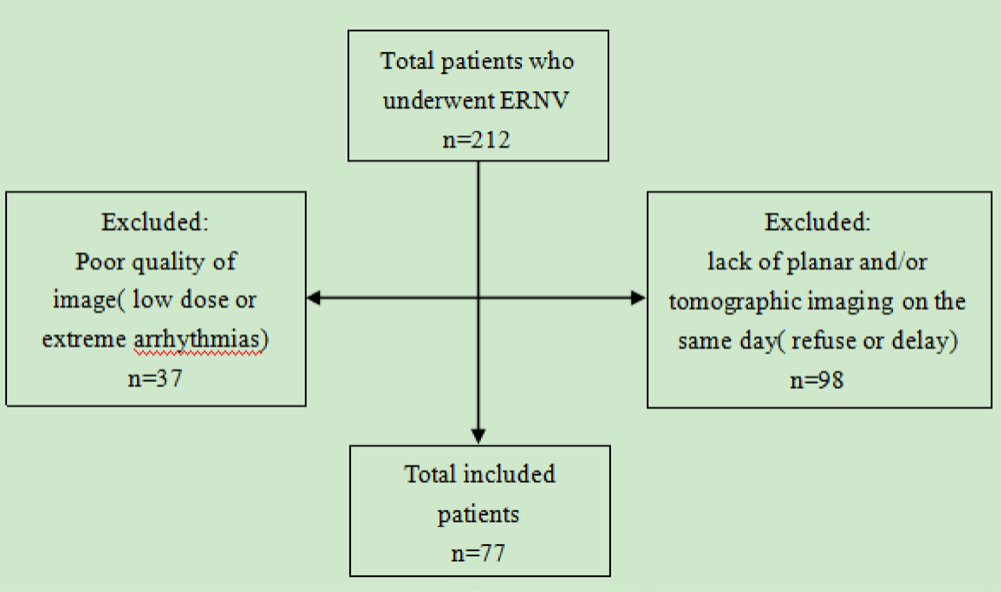
Flow chart of patients excluded from the study.

Two hundred twelve patients referred to our hospital and underwent ERNV from June 2018 to July 2022. Inclusion criteria: patients aged 18 to 90 years who underwent this examination and successfully completed it. Exclusion criteria: patients with extreme arrhythmias that could not be recognized by the cameras, and those who failed to complete planar and/or tomographic imaging on the same day (Fig. 1). Seventy-seven patients (49 males and 28 females) were finally included. They had at least 1 disease as: coronary heart disease, dilated cardiomyopathy, arrhythmia, or other cardiomyopathy and their LV/RV function and synchronization were needed clinically. All patients underwent planar imaging by C-SPECT followed tomographic imaging by CZT-SPECT on the same day. Two experienced nuclear medicine physicians analyzed the acquired data on the 2 cameras separately. The results were analyzed blindly. All patients were given informed consent, and the study was in accordance with the Declaration of Helsinki. The study protocol was approved by the Ethics Committee of TEDA International Cardiovascular Hospital (approval number: 2022-0429-4).

### 2.2. Acquisition of C-SPECT and CZT-SPECT imaging

The injected imaging agent was 99mTc to label autologous red blood cells by in vivo labeling method. The dose of radioactivity was about 740 to 925 MBq for each patient. About 15 to 20 minutes after injections, the patients lay on the bed of the C-SPECT (DiscoveryNM630, GE Healthcare, Milwaukee, WI) in the supine position. One of the detectors was rotated to the left anterior-oblique position of 45° and then slightly adjusted to the angle where the left and right ventricles were clearly separated. Then, the planar acquisition by the ECG gated-equilibrium method was performed. The acquisition matrix was 64 × 64. The energy window was ±15% and the energy peak was set at 140 keV. The R–R interval was divided into 16 equal parts. The heart rate window was ±15%. The total acquisition time was 15 minutes (about 6–8 million counts collected). Immediately after acquisition completion, the patients were transferred to CZT-SPECT (DiscoveryNM530c, GE Healthcare, Milwaukee, WI) scan room, and the detectors were closely attached to the patient’s left chest. After automatic recognition of cardiac blood pool shadow, the focal point was set in the center of the left ventricular (LV) cavity to start tomographic imaging. The acquisition time was 10 minutes. The other acquisition settings were same as the planar acquisition.

### 2.3. Imaging analysis

**Figure 2.**
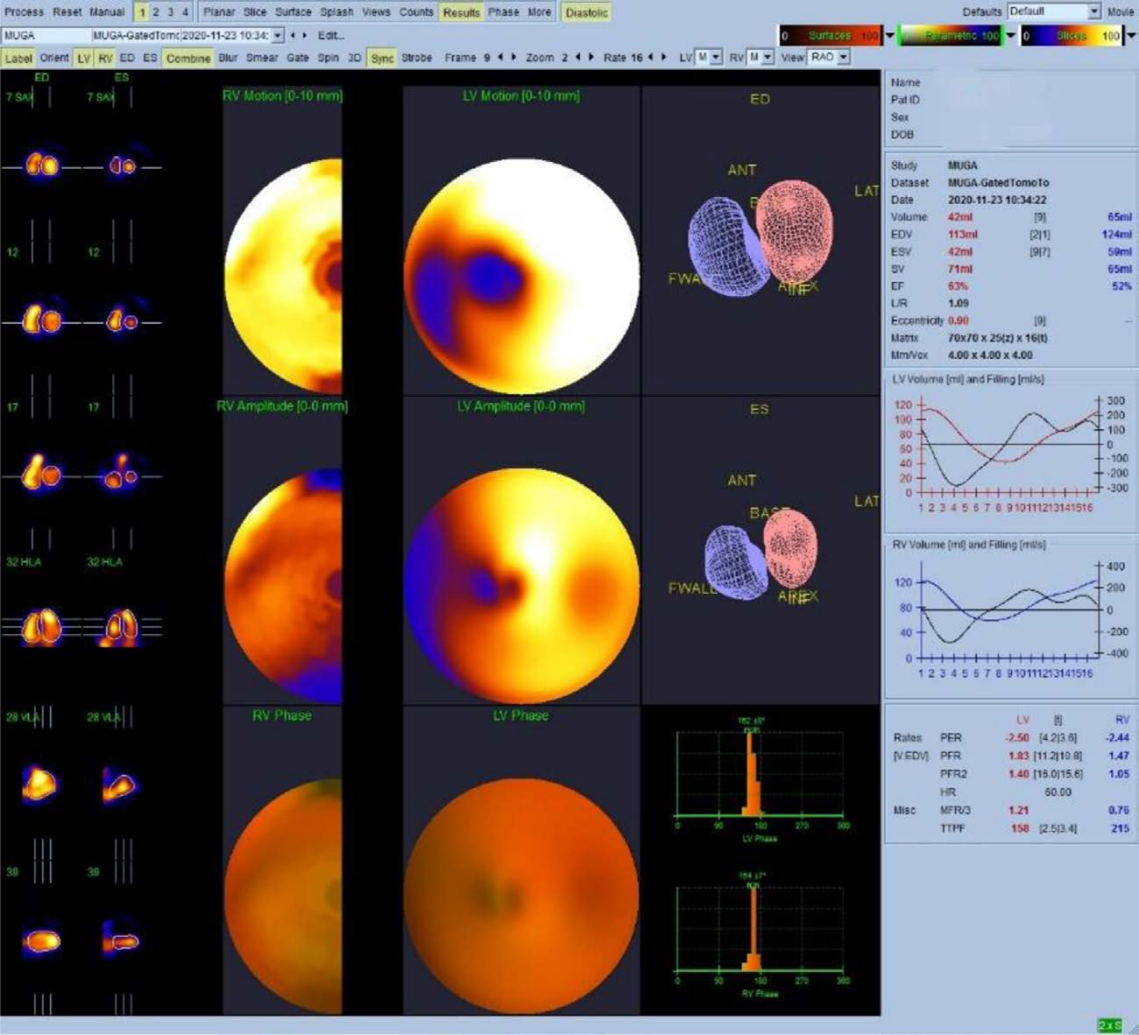
CZT-SPECT tomographic image and parameters calculated by Cedars-Sinai G-BPGs.

Data processing and recording were carried out at GE 4DR Xeleris Workstation, the planar imaging data were analyzed by ejection fraction (EF) analysis software (GE Healthcare, Milwaukee, WI), and the tomographic imaging data were analyzed by Cedars-Sinai G-BPGs software (Version2019, Cedars-Sinai, Ca) (Fig. 2).

### 2.4. Statistical methods

SPSS 26.0 statistical software was used for analysis. Normally distributed individuals were expressed as x ± s with paired *t* test and Pearson correlation analysis; non-normally distributed individuals were expressed as M (P25, P75) with Wilcoxon signed rank test and Spearman correlation analysis. Intraclass correlation coefficient (ICC) and Bland–Altman consistency analysis were applied for observer operational reproducibility evaluation. Correlation parameters were evaluated: 0.8 to 1.0 as excellent; 0.6 to 0.8 as good; 0.4 to 0.6 as normal; 0.2 to 0.4 as poor; <0.2 as no correlation.

## 3. Results

The characteristics of the patients were shown in Table 1. Twenty-one of the enrolled patients had CMR and ultrasound (US) findings during the same period (within 1 week).

**Table 1:** Basic characteristics of the patients.

Patient data	(n = 77)
Age (y)	61.3 ± 11.4
Male	49 (63.6%)
Female	28 (36.4%)
Height (cm)	167.7 ± 8.6
Weight (kg)	73.3 ± 15.1
Injection dose (MBq)	843.6 ± 85.1
Risk factor	
Hypertension	40 (51.9%)
Hyperlipemia	9 (11.7%)
Diabetes	19 (24.7%)
Smoking	24 (31.2%)
Drinking	12 (15.6%)
Medical/case history	
Myocardial infarction (MI)	13 (16.9%)
Pacemaker implantation	6 (7.8%)
Coronary stent	14 (18.2%)
Coronary artery by-pass grafting	3 (3.9%)
Clinical diagnosis	
Coronary heart disease (CAD)	33 (42.9%)
Dilated cardiomyopathy (DCM)	26 (33.8%)
Arrhythmia	26 (33.8%)
Others (valvular disease, rheumatic heart disease, etc)	3 (3.9%)

### 3.1. Comparison of LVEF and RVEF between 2 cameras

**Figure 3.**
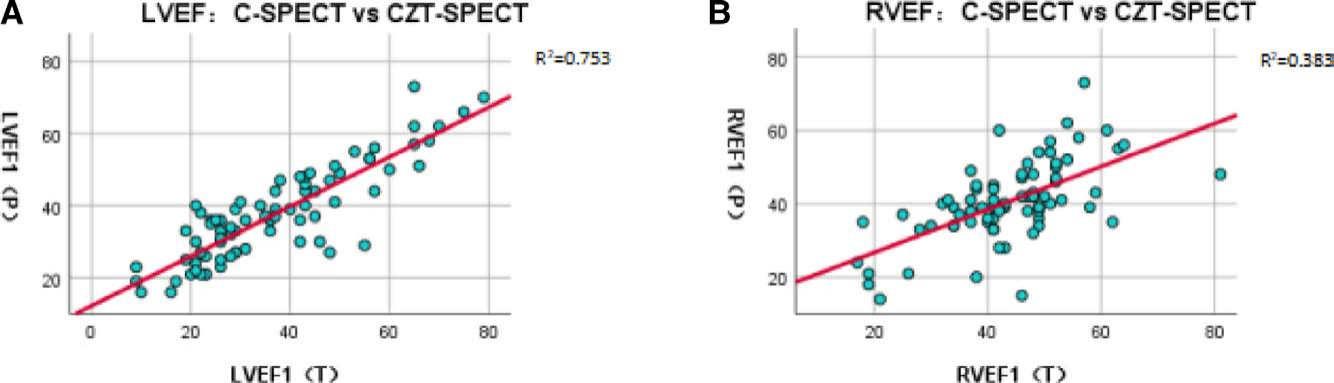
Scatter diagram of LVEF/RVEF between CZT-SPECT and C-SPECT.

The functional parameters obtained by planar imaging were added with the suffix (P) (abbreviation for Planar) and those obtained by CZT-SPECT tomography with the suffix (T) (abbreviation for Tomography) for differentiation. As shown in Table 2 and Figure 3, LVEF (P) = 37.8 ± 13.0% and LVEF (T) = 35.0% (24.0, 48.0), respectively, with no significant difference (*P* > .05) and excellent correlation (*R* = 0.831, *P* < .001) between them.

**Table 2:** Comparison of LVEF and RVEF between C-SPECT and CZT-SPECT.

Statistical parameters and values	LVEF (P)	LVEF (T)	RVEF (P)	RVEF (T)
Mean ± SD/M (P25, P75)%	37.8 ± 13.0	35.0 (24.0, 48)	40.6 ± 10.8	43.7 ± 11.4
Mean (95% CI)%	37.8 (34.8, 40.7)	37.1 (33.4, 40.8)	40.6 (38.2, 43.1)	43.7 (41.1, 46.3)
*P* value*	.245		.007	
*R* value (correlation)*	0.831 (*P* = .000)		0.619 (*P* = .000)	

CI = confidence interval, LVEF = left ventricular ejection fraction, RVEF = right ventricular ejection fraction, SD = standard deviation.

* LVEF (P)/RVEF (P) comparing with LVEF (T)/RVEF (T).

The RVEF provided by the 2 cameras were RVEF (P) = 40.6 ± 10.8% and RVEF (T) = 43.7 ± 11.4%, respectively, with significant difference (*P* < .05) and good correlation (*R* = 0.619, *P* < .001).

### 3.2. Comparison with CMR results

**Figure 4.**
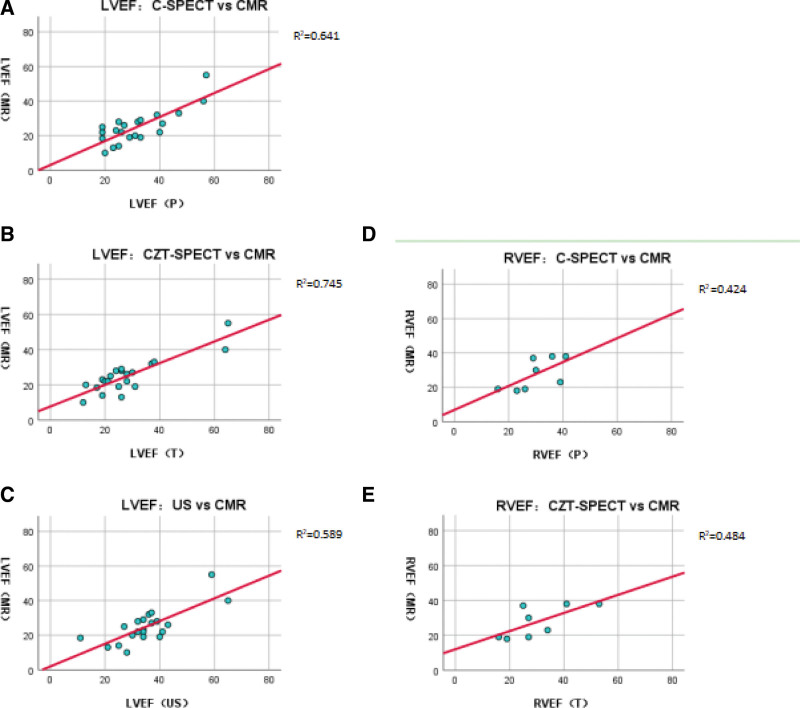
Scatter diagram of the results compared with CMR. (a, b, and c) are LVEF comparisons of C-SPECT, CZT-SPECT, and US with CMR, respectively; (d and e) are RVEF comparisons of C-SPECT and CZT-SPECT with CMR, respectively.

Twenty-one of 77 patients had LVEF (MR) and LVEF (US). Eight of this 21 had RVEF (MR). As shown in Figure 4 and Table 3: LVEF (P) = 29.0% (23.5, 39.5), LVEF (T) = 26.0% (19.5, 30.5), LVEF (US) = 35.2 ± 11.5%, and LVEF (MR) = 23.0% (19.0, 28.5). There was significant difference between LVEF (P) and LVEF (MR) (*P* < .05), as well as between LVEF (US) and LVEF (MR) (*P* < .001). The correlation was both good (*R* = 0.660, *P* < .05 and *R* = 0.658, *P* < .05). There was no significant difference between LVEF (T) and LVEF (MR) (*P* > .05). The correlation was good (*R* = 0.695, *P* < .001).

**Table 3:** Comparison with CMR results (LVEF/RVEF of C-SPECT and CZT-SPECT, LVEF of US).

Statistical parameters and values	LVEF (P)	LVEF (T)	LVEF (US)	LVEF (MR)	RVEF (P)	RVEF (T)	RVEF (MR)
Mean ± SD/M (P25, P75)%	29.0 (23.5, 39.5)	26.0 (19.5, 30.5)	35.2 ± 11.5	23.0 (19.0, 28.5)	30.0 ± 8.5	30.3 ± 12.1	26.5 (19.0, 37.8)
Mean (95% CI)%	31.7 (26.5, 36.8)	28.1 (21.9, 34.4)	35.2 (29.9, 40.4)	25.0 (20.5, 29.5)	30.0 (22.9, 37.1)	30.3 (20.1, 40.4)	27.8 (20.2, 35.3)
*P* value*	.001	.085	.0001		.446	.482	
*R* value (correlation)*	0.660 (*P* = .001)	0.695 (*P* = .0005)	0.658 (*P* = .001)		0.771 (*P* = .025)	0.745 (*P* = .034)	

CI = confidence interval, LVEF = left ventricular ejection fraction, RVEF = right ventricular ejection fraction, SD = standard deviation.

* LVEF (P)/LVEF (T)/LVEF (US) comparing with LVEF (MR); RVEF (P)/RVEF (T)/RVEF (US) comparing with RVEF (MR).

RVEF (P) = 30.0 ± 8.5%, RVEF (T) = 30.3 ± 12.1%, and RVEF (MR) = 26.5% (19.0, 37.8). There was no significant difference between RVEF (P) and RVEF (MR) (*P* > .05), as well as between RVEF (T) and RVEF (MR) (*P* > .05). The correlation was good (*R* = 0.771, *P* < .05 and *R* = 0.745, *P* < .05).

### 3.3. Comparison of LVPER (peak ejection rate [PER]), LVPFR (peak filling rate [PFR]), RVPER, and RVPFR between the 2 models

**Figure 5.**
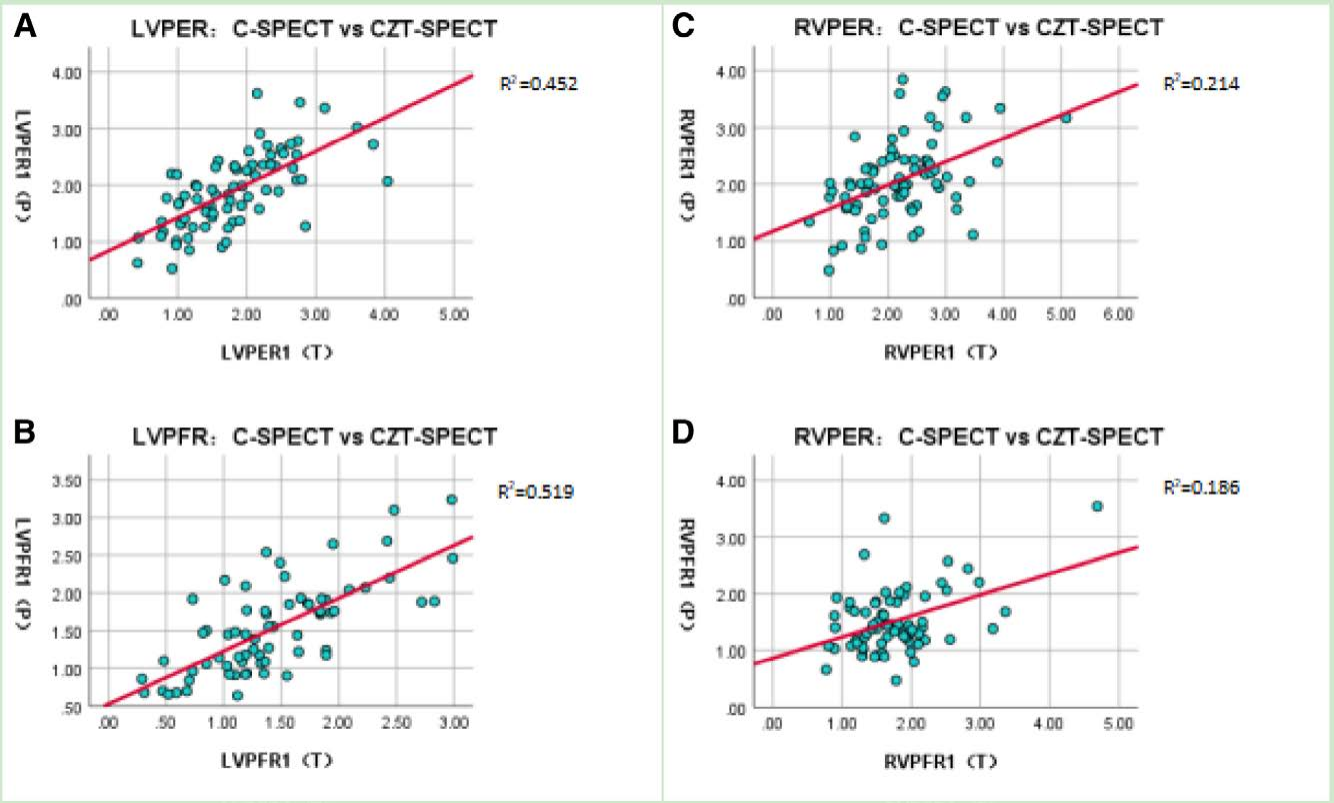
Scatter diagram of LVPER, LVPFR, RVPER, and RVPFR between CZT-SPECT and C-SPECT.

LVPFR (T) was not available in 8 cases and RVPFR (T) was not available in 5 cases, which were not included in the analysis. As shown in Table 4 and Figure 5, LVPER (P) = 1.90 ± 0.66 and LVPER (T) = 1.81 ± 0.76, respectively, with no significant difference (*P* > .05) and good correlation (*R* = 0.672, *P* < .001). LVPFR (P) = 1.47 (1.07, 1.90) and LVPFR (T) = 1.43 ± 0.62, with significant difference (*P* > .05) and good correlation (*R* = 0.700, *P* < .001).

**Table 4:** Comparison of LVPER, LVPFR, RVPER and RVPFR between the 2 cameras.

Statistical parameters and values	LVPER (P)	LVPER (T)	LVPFR (P)	LVPFR (T)	RVPER (P)	RVPER (T)	RVPFR (P)	RVPFR (T)
Mean ± SD/M (P25, P75)	1.90 ± 0.66	1.81 ± 0.76	1.47 (1.07, 1.90)	1.43 ± 0.62	2.07 ± 0.70	2.19 ± 0.79	1.40 (1.16, 1.85)	1.65 (1.35, 1.99)
Mean (95% CI)	1.90 (1.75, 2.05)	1.81 (1.64, 1.99)	1.53 (1.39, 1.68)	1.43 (1.28, 1.58)	2.07 (1.91, 2.23)	2.19 (2.01, 2.37)	1.52 (1.39, 1.65)	1.76 (1.61, 1.91)
*P* value*	.180		.101		.173		.0009	
*R* value (correlation)*	0.672 (*P* = .000)		0.700 (*P* = .000)		0.463 (*P* = .00002)		0.253 (*P* = .032)	

CI = confidence interval, PER = peak ejection rate, PFR = peak filling rate, SD = standard deviation.

* LVPER (P)/LVPFR (P)/RVPER (P)/RVPFR (P) comparing with LVPER (T)/LVPFR (T)/RVPER (T)/RVPFR (T).

RVPER (P) = 2.07 ± 0.70 and RVPER (T) = 2.19 ± 0.79, respectively, with no significant difference (*P* > .05) but normal correlation (*R* = 0.463, *P* < .001). RVPFR (P) = 1.40 (1.16, 1.85) and RVPFR (T) = 1.65 (1.35, 1.99), with significant difference (*P* < .05) and poor correlation (*R* = 0.253, *P* < .05).

### 3.4. Repeatability comparison

**Figure 6.**
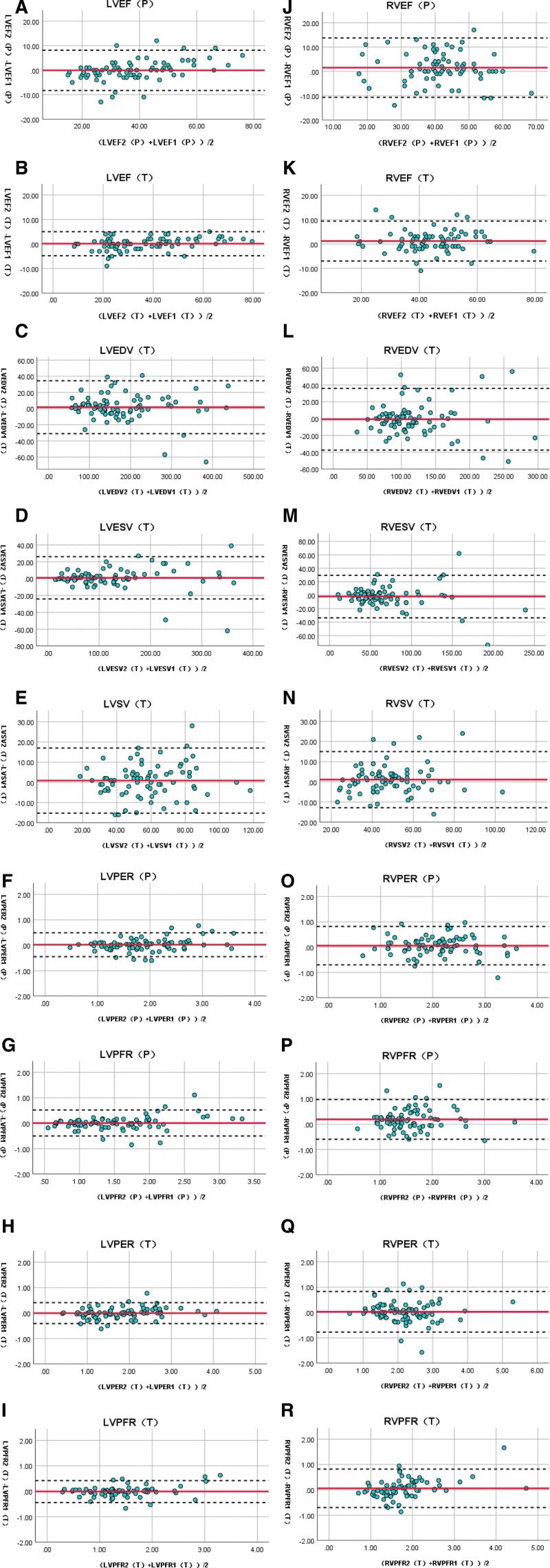
Bland–Altman analysis diagram of the repeatability of each parameter measurement. (a), (b) and (j), (k) are the repeatability analyses of LVEF and RVEF obtained by C-SPECT and CZT-SPECT, respectively; (c)–(e) and (l)–(n) are the repeatability analyses of LV/RV volume parameters obtained by CZT-SPECT, respectively; (f)–(i) and (o)–(r) are the repeatability analyses of LV/RV PER and PFR obtained by C-SPECT and CZT-SPECT, respectively.

The data of observer 1 and observer 2 were shown in Table 5. The Bland–Altman consistency analysis evaluating interobserver reproducibility deviations was shown in Figure 6.

**Table 5:** Interobserver reproducibility analysis.

LVEF and LV volume parameters					
Groups, statistical parameters, and values	LVEF (P) (%)	LVEF (T) (%)	LVEDV (mL)	LVESV (mL)	LVSV (mL)
Observer 1	37.8 ± 13.0	35.0 (24.0, 48.0)	158.0 (116.5, 220.0)	100.0 (61.0, 157.5)	57.5 ± 20.3
Observer 2	36.0 (25.0, 47.5)	33.0 (23.5, 47.5)	163.0 (117.5, 226.0)	102.0 (64.5, 158.5)	58.4 ± 21.1
Correlation coefficient (95% Cl)*	0.956 (0.931, 0.972)	0.989 (0.983, 0.993)	0.984 (0.974, 0.990)	0.989 (0.983, 0.993)	0.921 (0.878, 0.949)
Mean difference	−0.1	0.1	1.7	1	0.9
Standard deviation	4.2	2.5	16.7	12.8	8.2
Upper limit of 95% confidence interval	8.132	5	34.432	26.088	16.972
Lower limit of 95% confidence interval	-8.332	−4.8	−31.032	−24.088	−15.172
CV (%)	11.1	6.7	6.1	10.2	14.1

RVEF and RV volume parameters					
Groups, statistical parameters, and values	RVEF (P) (%)	RVEF (T) (%)	RVEDV (mL)	RVESV (mL)	RVSV (mL)
Observer 1	40.6 ± 10.8	43.7 ± 11.4	103.0 (83.0, 140.0)	59.0 (43.5, 82.0)	46.0 (36.0, 56.5)
Observer 2	42.12 ± 10.3	44.8 ± 11.4	106.0 (82.5, 137.5)	58.0 (43.0, 79.0)	49.0 (38.0, 57.5)
Correlation coefficient (95% Cl)*	0.823 (0.732, 0.884)	0.929 (0.887, 0.955)	0.933 (0.897, 0.957)	0.926 (0.886, 0.952)	0.899 (0.846, 0.934)
Mean difference	1.5	1.2	−0.8	−2	1.1
Standard deviation	6.2	4.2	18.7	16.1	7.1
Upper limit of 95% confidence interval	13.652	9.432	35.852	29.556	15.016
Lower limit of 95% confidence interval	−10.652	−7.032	−37.452	−33.556	−12.816
CV (%)	14.9	9.5	16.0	23.4	14.7

LVPER and LVPFR					
Groups, statistical parameters, and values	LVPER (P)	LVPFR (P)	LVPER (T)		LVPFR (T)
Observer 1	1.90 ± 0.66	1.45 (1.05, 1.89)	1.81 ± 0.76		1.43 ± 0.62
Observer 2	1.92 ± 0.74	1.40 (1.02, 1.87)	1.69 (1.11, 2.45)		1.32 (1.01, 1.86)
Correlation coefficient (95% Cl)*	0.940 (0.907, 0.961)	0.912 (0.865, 0.943)	0.964 (0.944, 0.977)		0.943 (0.909, 0.965)
Mean difference	0.02	0.006	−0.003		−0.007
Standard deviation	0.24	0.26	0.21		0.22
Upper limit of 95% confidence interval	0.4904	0.5156	0.4086		0.4242
Lower limit of 95% confidence interval	−0.4504	−0.5036	−0.4146		−0.4382
CV (%)	12.6	17.2	11.6		15.7

RVPER and RVPFR					
Groups, statistical parameters, and values	RVPER (P)	RVPFR (P)	RVPER (T)		RVPFR (T)
Observer 1	2.07 ± 0.70	1.38 (1.13, 1.79)	2.19 ± 0.79		1.65 (1.35, 2.00)
Observer 2	2.12 ± 0.66	1.59 (1.28, 1.98)	2.22 ± 0.76		1.77 (1.29, 2.15)
Correlation coefficient (95% Cl)*	0.838 (0.757, 0.894)	0.698 (0.507, 0.814)	0.860 (0.789, 0.909)		0.857 (0.780, 0.909)
Mean difference	0.06	0.19	0.03		0.06
Standard deviation	0.39	0.4	0.41		0.39
Upper limit of 95% confidence interval	0.8244	0.974	0.8336		0.8244
Lower limit of 95% confidence interval	−0.7044	−0.594	−0.7736		−0.7044
CV (%)	18.6	25.3	18.6		21.7

CV = coefficient of variation, EDV = end-diastolic volume, ESV = end-systolic volume, SV = stroke volume.

* All *P* value of correlation coefficient approached 0.

The ICCs for LVEF/RVEF of 2 cameras, and LV/RV volume parameters were excellent (0.823–0.989, all *P* < .001). The proportion outside the Bland–Altman consistency analysis 95% confidence intervals ranged from 5.2% to 10.4%.

Data were not available for observer 1 and/or observer 2 in 11 cases for LVPFR (T) and 8 cases for RVPFR (T), which were not included in the analysis. ICC for RVPFR (P) was good (0.698, *P* < .001). ICC for LVPER, LVPFR of both cameras, PER (P), PER (T), and PFR (T) of RV were excellent (0.838–0.964, all *P* < .001). The proportion outside the Bland–Altman consistency analysis 95% confidence intervals ranged from 4.3% to 9.1%.

## 4. Discussion

EF is one of the most important indicators of cardiac function. Its gold standard is ventriculography, which is invasive and costly. In contrast, the commonly accepted gold standard of the noninvasive method is CMR, which is limited in clinical use because of its complicated techniques and many contradictions. 99mTc-RBC ERNV is a noninvasive exam to measure the change of blood volume in the ventricles. It best fits the concept of EF. Tomographic imaging allows the LV/RV to be reconstructed in a 3D mode which more closely resembles the complete standard measurement.

In this study, the LVEF correlation was excellent for 2 cameras and the RVEF correlation was good but lower than LVEF (*R* value: 0.619 vs 0.831). This is in agreement with most of the previous studies.^[15–17]^ Chen YC et al^[17]^ concluded that the reason for this situation is the inaccuracy of the algorithm in CZT-SPECT. The influence of LV size and shape in the identification of RV boundaries by the QBS software leads to the reduced correlation with the planar method. Although this may be part of the reasons, we believe that the aforementioned effects in planar C-SPECT are more evident. Moreover, the difficulty in identifying the location of the pulmonary artery trunk also affects the outline of the RV basal part in C-SPECT planar rather than CZT-SPECT tomography.

As CMR the gold standard, the statistical analysis results of C-SPECT and US were very similar in terms of LVEF, while the results of CZT-SPECT were better. In comparison with previous studies,^[7,8]^ we obtained a lower correlation between LVEF (T) and LVEF (MR) (*R* value: 0.695 vs 0.92 and 0.89). This is most likely related to the selection of the samples. There are wider range of disease types in our cases, especially heart failure and arrhythmias (especially atrial fibrillation and complete left bundle branch block), which may significantly affect the results. Both RVEF (P) and RVEF (T) were not significantly different from RVEF (MR). The correlation was good (0.771 and 0.745, respectively) and better than the results of LVEF (P)/LVEF (T) with LVEF (MR). Previous studies comparing tomographic ERNV with CMR have concluded a good correlation between the 2, both in terms of RVEF and RV volume,^[7,8,16,18,19]^ but the studies differed significantly (*R* value: 0.41–0.81). The reason probably was the large differences in the selection of samples in each study. For example, the study of Xie BQ^[8]^ included all patients with dilated cardiomyopathy, but ischemic cardiomyopathy in the study of Anderson K,^[18]^ while Apert A^[19]^ selected patients with multiple cardiomyopathies, but excluded patients with arrhythmias. The type of disease included in our study was relatively broad, but the sample size was too small. Based on this, our results suggest that CZT-SPECT tomographic ERNV may be comparable to CMR in measuring RVEF in patients with multiple diseases, but we also need large more samples to support.

There are very few studies on PER and PFR in CZT-SPECT tomographic ERNV. On the one hand, there is no accepted “gold” standard for these 2 parameters. On the other hand, CZT-SPECT myocardial perfusion imaging can also obtain several functional parameters such as PER and PFR of LV. Myocardial perfusion imaging is more convenient than ERNV, which has been studied more in recent years,^[20,21]^ especially the systolic and diastolic function of LV under stress.^[22]^ Studies on PER and PFR of RV are rare in available examination techniques. In our study, LVPER and LVPFR correlated well for 2 cameras (*R* values: 0.672 and 0.700), but less well compared to LVEF. This is within our expectations. LVEF is obtained from 2-frame images based on end-diastolic volume (EDV) and end-systolic volume (ESV) target areas. In comparison, LVPER and LVPFR are obtained from the count (volume)–time curves based on 16-frame reconstructions, which may cause a larger cumulative deviation. RVPER and RVPFR correlations we obtained for 2 cameras were only 0.463 and 0.253, with statistically significant difference in RVPFR. This is significantly lower than our expectations. We analyze further to try to find some possible reasons or the regularity of this bias, but obtain no meaningful results. We found significant uncertainty in the deviation of 2 cameras in measuring RVPER and RVPFR. In some case the higher values were measured by C-SPECT, and in others by CZT-SPECT. The numbers of 2 were close and deviation values were larger, which were particularly evident in RVPFR. This may indicate that we need a third-party comparison. Before that, we are not sure if this low correlation related to our sample selection, so we think it may first need a large normal group of samples for control.

In terms of repeatability, the intraclass correlation coefficient of RVPFR (P) was good (ICC = 0.698), while those of other groups of data was excellent (ICC: 0.823–0.989). This was similar to previous studies.^[13,15,17,23]^ Using Bland–Altman diagram analysis, the confidence intervals for the repeatability of both LVEF and RVEF of CZT-SPECT were narrower than those of C-SPECT respectively, indicating that the repeatability of CZT-SPECT was better. The intervals for the repeatability of LVEF for both cameras were narrower than those for the corresponding RVEF respectively. It indicated that the repeatability of LVEF measurement is better for both cameras than RVEF. The confidence intervals for repeatability of LVPER and LVPFR of the 2 cameras did not differ much in width. The LVPER group was slightly better than the corresponding LVPFR group respectively, while similar results were obtained for RVPER and RVPFR. The confidence intervals for repeatability of PER (P), PFR (P), PER (T), and PFR (T) of LV were significantly narrower than those of RV, indicating that the repeatability of LVPER and LVPFR of 2 cameras was all better than that of RV.

In this study, we introduced the coefficient of variation (CV). It is a measure of reproducibility applied in previous studies by Jensen et al,^[15,23]^ with higher values indicating poorer reproducibility. We obtained the same trends in LV/RV EF and volume parameters as in the study by Jensen et al,^[15]^ but slightly higher values. It might be related to the selection of the case sample. They selected cancer patients without cardiovascular diseases, whose cardiac function were relatively normal. In contrast, our sample consisted entirely of patients with various types of cardiovascular diseases, most of them having heart failure. In our results, the CV values of LVEF (P), LVEF (T), RVEF (P), and RVEF (T) were 11.1%, 6.7%, 14.9%, and 9.5%, respectively. In the CZT-SPECT group, the CV value was significantly lower than in the C-SPECT group and in the LVEF group was lower than in the corresponding RVEF group, respectively. The above was also indicated in the Bland–Altman diagram. Even we can see that the CV value of RVEF (T) were lower than that of LVEF (P), indicating that CZT-SPECT was significantly better than C-SPECT overall in terms of interobserver repeatability. The CV values of LVEDV (T), LVESV (T), LVSV (T), RVEDV (T), RVESV (T), and RVSV (T) were 6.1%, 10.2%, 14.1%, 16.0%, 23.4%, and 14.7%, respectively. The CV values of LV measurements were all significantly lower than those of the corresponding RV measurements, indicating that the reproducibility of LV volume parameters measurements was significantly better than RV. The first reason is the problem of RV boundary recognition, especially the right atrioventricular division and pulmonary artery trunk location, as mentioned above. It has always been a problem in cardiac blood pool imaging since planar to tomography. Another reason is that the repeatability for small heart chambers is worse than that for larger chambers, which was also mentioned in Jensen et al’s study. Most of the samples in our study had normal right heart function, and some right heart chambers were relative smaller. In addition, most of these samples had serious cardiovascular diseases with LV involvement and enlarged heart chambers. The ratio of left and right cardiac chambers was increased. The RV recognition was more obviously affected by the enlarged left ventricle, which further affected the repeatability of RV measurement. However, the effects for RVEDV and RVESV were in a close tend, so that the CV values of RVSV and LVSV were very close. It explained why the repeatability of RVEF (T) was not significantly different from LVEF (T).

CV values of the PER group were better than the PFR group, also better in the LV group than in the RV group. It is worth noting that LVPFR (T) and RVPFR (T) in some cases could not be obtained, which were mentioned in Sections 3.3 and 3.4. This had not happened in any other data analysis. The software identifies it as NA, which meant unrecognized or invalid data. Neither previous studies nor software manuals have mentioned the reason for such situation and we could not exactly explain these reasons either. By observing and analyzing the volume (and filling)–time curve of each sample, we tried to find the potential reasons to explain the NA data emerged. We preferred 1 possibility based on distinguishing the early peak filling rate (PFR) and late peak filling rate (PFR1) by BPGs software. In the volume (and filling)–time curve, BPGs recognized the maximum negative value of the filling curve, which obtained from the volume curve, as PER (the deepest trough in the filling curve), then the following 2 positive peaks (peak 1 and peak 2) would be further identified as the value points of PFR and PFR1 (or only identified PFR if only single peak). We found that most of these NA data have the following characteristic: there was a trough with negative value between peak 1 and peak 2, and the absolute value of this trough was not much different from peak 1 or significantly larger than peak 1. In the few of these NA data, there might be another characteristic: peak 1 and peak 2 were not significantly different in absolute value, and there was a less obvious trough with positive value between them. The cause of this wave form of the filling curve should be related to the sample we selected, based on the waveform of the volume curve disorder caused by significant impairment of ventricular diastolic function in patients with severe cardiovascular disease. It was our observation and analysis, but not a scientific argument. We described the phenomenon, but cannot draw conclusion. However, we proved that this defect exists by our data. This situation may suggest that PFR obtained by CZT-SPECT is not a stable parameter for monitoring ventricular diastolic function. By comparison, PER is much more stable and has better reproducibility than PFR as motioned above. However, previous studies have shown that diastolic impairment of the ventricle precedes systolic impairment.^[24]^ We need to further investigate BPGs or other software to obtain PFR stably.

## 5. Limitations

The relatively small number of cases for which concurrent CMR data were available, especially for RV function. There was no “gold” standard technical reference for diastolic function, as well as noncardiac normal controls due to ethical constraints. As far as we know, this study includes the broadest type of cardiovascular diseases among the published studies on CZT-SPECT tomographic ERNV. It may illustrate the generalizability of CZT-SPECT tomographic ERNV to patients with cardiovascular disease, but more sample sizes are needed.

## 6. Conclusion

CZT-SPECT tomographic ERNV has good correlation with C-SPECT planar ERNV in the evaluation of LV/RV systolic function and LV diastolic function. It correlates good with CMR in the evaluation of LV/RVEF and its reproducibility is significantly better than that of C-SPECT. The determination of RV diastolic function requires further study. In view of the excellent temporal, spatial and energy resolution of CZT-SPECT and the high detection efficiency of photon counting, CZT-SPECT tomographic ERNV will play an important and unique role in the clinical application of accurate evaluation of biventricular function in the future.

## Author contributions

**Conceptualization:** Yue Chen, Jianming Li.

**Formal analysis:** Zekun Pang, Jiao Wang, Xuewen Yang.

**Investigation:** Zekun Pang.

**Writing – original draft:** Yue Chen.

**Writing – review & editing:** Jianming Li.
